# Supplementary material for: Mesenchymal stem cell-derived extracellular matrix for musculoskeletal tissue regeneration
Source: Commun Biol. 2026 Jan 31;9:147. doi: 10.1038/s42003-026-09638-3 (PMC12860798; doi:10.1038/s42003-026-09638-3)
Supplement: Supplementary file 1 — Supplementary Information [file 42003_2026_9638_MOESM1_ESM.pdf]

Supplementary Table 1. Advantages and disadvantages of ECM from different sources

dECM origin	**advantages**	**disadvantages**
assessment of their mature cell (eg. chondrocyte)	1. Easily available;	1. Supply limitations;<br>2. Donor morbidity and pain are difficult to avoid;<br>3. Easy to dedifferentiate[1,2].
tissue[3] (eg. articular cartilage)	1. Easily available;	1. Expensive;<br>2. Animal-derived;<br>3. Large batch variation between donors
mECM	1. Preserve the characteristics of seed cells better compared to other ECM materials;<br>2. Autologous materials;<br>3. Reduce the likelihood of adverse immune reactions and the spread of pathogens[4].	1. Necessitates substantial quantities of donor cells to produce dECM;<br>2. Suffers from batch-to-batch variations;<br>3. Manufacturing a large amount of dECM is challenging[5].

Supplementary Table 2. Different decellularization methods of mECM

Method Category	Specific Protocol	MSCs Source	Key Findings / Outcome
Biological/Chemical	0.5% SDS+1%Triton X-100 + DNase	BM-MSCs	It has good biocompatibility, is non-cytotoxic, and can promote the osteogenic and chondrogenic differentiation of BM-MSCs[6].
Physical/Chemical	cryogenic milling + freeze-drying + dehydrothermal	BM-MSCs	Effective DNA removal; preserved glycosaminoglycan (GAG)[7] content and nano-fibrous structure; promoted chondrogenic differentiation.
Physical/Chemical	Freeze-Thaw cycles + 25 mM NH_4OH	MSCs	Nucleus was completely removed, with extremely low residual DNA (4.38 ± 1.67 ng), and the ECM structure was intact[8].
Chemical	0.1% Triton X-100 + 1.5M KCl	MSCs	Nucleus was completely removed, with extremely low residual DNA (7.24 ± 2.15 ng), and the ECM structure was intact[8].
Biological/Chemical	0.5% Triton X-100 + 20 mM NH_4OH + DNase	UC-MSCs	No cell nucleus residue, promoting the survival of neurons and axon growth[9].

Supplementary Table 3. Applications of mECM for bone regeneration

mECM origin	Seeded cell types and animal models	mECM Processing Methods	Function
Mouse BM-MSCs	Mouse BM-MSCs and nude mice	/	Facilitated expansion of mesenchymal colony-forming units *in vitro* while preserving stem cell properties[10]
Human BM-MSCs	Human BM-MSCs and nude mice	/	Strongly promoted proliferation; retained stem cell properties with a low level of ROS[11]
Human BM-MSCs	Human BM-MSCs	/	Strongly promoted proliferation; increased osteogenic potential; decreased intracellular ROS[12]
Human BM-MSCs	Human BM-MSCs	/	Significantly enhanced the osteoinductive and antioxidant effects of melatonin[13]
Human UC-MSCs	Human UC-MSCs	/	Facilitated cell proliferation; preserved the osteogenic differentiation potential by enhancing resistance to oxidative stress-induced premature senescence[14]
Human UC-MSCs	Human UC-MSCs	/	Promoted proliferation; improved resistance to exogenous oxidative stress; preserved the osteogenic differentiation potential[15]
Mouse BM-MSCs	BMMs	/	Significantly suppressed osteoclastogenesis via the attenuation of intracellular ROS[16]
Rat BM-MSCs	Rat BM-MSCs and Rat	mECM as a scaffold	Increased osteogenic potential; promoted osseointegration *in vitro* and *in vivo*[17]
Rabbit BM-MSCs	Rabbit BM-MSCs and knee joint osteochondral defect model in rabbits	mECM as a scaffold	Increased osteogenic potential; significantly improved the regeneration of osteochondral defects in rabbits[6]
Human BM-MSCs	Human BM-MSCs and nude mice	mECM as a scaffold coating	Could be used as a delivery platform for BMP-2; promoted ectopic bone formation in mice[18]

mECM origin	Seeded cell types and animal models	mECM Processing Methods	Function
Human BM-MSCs	Human BM-MSCs and nude mice	mECM as a scaffold coating	Increased osteogenic potential; induced the formation of a mineralized matrix *in vivo*[19]
Human BM-MSCs	Human BM-MSCs and nude mice	mECM as a scaffold coating	Induced more calcium deposition and VEGF *in vitro*; promoted blood vessel and bone formation *in vivo*[20]
Rat BM-MSCs	Rat BM-MSCs and Rat	mECM as a scaffold	Retained BMP-2 and TGF-β1 and high osteoinductive and osteoconductive capacity[21]
Human BM-MSCs	Human BM-MSCs and nude mice	mECM as a scaffold coating	Induced MSCs to secrete more proangiogenic factors while maintaining the markers of osteogenic differentiation; enhanced cellularization and osteogenic differentiation *in vivo*[22]
Rat BM-MSCs	Rat BM-MSCs	mECM as a scaffold coating	The poly(epsilon-caprolactone) PCL/ECM composite scaffolds containing the most mature mineralized matrix had the strongest ability to promote osteogenic differentiation[23]
Rat BM-MSCs	Rat BM-MSCs	mECM as a scaffold coating	Maintained osteogenic differentiation in the absence of dexamethasone[24]
Human UC-MSCs	THP-1 Cells and nude mice	mECM as a scaffold coating	Attenuated the foreign body response; improved bone regeneration by increasing the accumulation of M2 macrophages[25]
Human BM-MSCs	Human BM-MSCs and nude mice	mECM as a scaffold coating	Promoted cell proliferation; retained ‘‘stemness’’; promoted bone formation *in vivo*[26]

Supplementary Table 4. Applications of mECMs for cartilage regeneration

mECM origin	Seeded cell types and animal models	mECM Processing Methods	Function
Human BM-MSCs	Human BM-MSCs	mECM as a Scaffold	Supported cell adhesion; the early stage chondrogenesis-mimicking-ECM scaffold facilitated chondrogenesis[27]
Human BM-MSCs	Human BM-MSCs	milled mECM for scaffold fabrication	Enhanced chondrogenesis[7]
Human BM-MSCs	Human IPFSC	milled mECM for scaffold fabrication	Enhanced chondrogenesis[28]
Human BM-MSCs	Human BM-MSCs	/	Enhanced cell proliferation; enhanced chondrogenic potential[12]
Human BM-MSCs (fetal and adult)	Human BM-MSCs	/	Fetal MSCs ECM retarded the senescence and enhanced chondrogenesis of late-passage adult MSCs[29]
Human BM-MSCs	Human BM-MSCs	mECM as a Scaffold	Promoted cell proliferation; dECM from BM-MSCs had a more pronounced effect on chondrogenic gene expression than dECM from chondrocytes[30]
Human IPFSC	Human IPFSC	/	dECM with 50μM and 250μM of L-ascorbic acid phosphate treatment significantly promoted chondrogenesis[31]
Human SM-MSCs	Human SM-MSCs (passage 8)	/	Enhanced chondrogenesis[32]
Human SM-MSCs	Human SM-MSCs	/	Enhanced adult human SM-MSCs *in vitro* expansion and chondrogenic potential; promoted expanded cell antioxidation ability[33]
Human SM-MSCs (fetal and adult)	Human adult SM-MSCs	/	Fetal SM-MSCs ECM was superior to adult SM-MSCs ECM in promoting cell proliferation and chondrogenic potential[34]
Human SM-MSCs	Human SM-MSCs	/	Enhanced chondrogenesis[35]

mECM origin	Seeded cell types and animal models	mECM Processing Methods	Function
Human SM-MSCs	Human SM-MSCs	/	sb203580 preconditioning enhanced dECM expanded human SM-MSCs proliferation and chondrogenic potential; sb203580 preconditioning promoted dECM rejuvenated human SM-MSCs' ability against inflammation during chondrogenic induction[36]
Porcine SM-MSCs	Porcine SM-MSCs	/	Greatly enhanced chondrogenic capacity[37]
Porcine SM-MSCs	Porcine SM-MSCs	/	Greatly enhanced SM-MSCs proliferation; enhanced Chondrogenesis; Inhibited cell hypertrophy[38]
Porcine SM-MSCs	Porcine SM-MSCs and minipigs	/	Promoted cell proliferation; enhanced expanded cell Chondrogenic potential *in vitro* and *in vivo*[39]
Porcine SM-MSCs and IPFSC	Porcine IPFSC	/	Significantly enhanced chondrogenesis with a lower level of intracellular ROS; Inhibited cell hypertrophy[40]
Rabbit BM-MSCs	Rabbit BM-MSCs and rabbits	/	Induced chondrogenic differentiation; repaired articular cartilage *in vivo*[41]
Human IPFSC	Human IPFSC and nude mice	/	dECM that derived from SV40LT transduced infrapatellar fat pad-derived stem cells enhanced enhanced chondrogenesis *in vitro* and *vivo*[42]
Rabbit BM-MSCs and porcine BM-MSCs	Rabbits and minipigs	mECM as a Scaffold	Increased the number of bone MSCs and improved the extent of cartilage repair *in vivo*[43]
Rabbit BM-MSCs	Rabbit chondrocyte and nude mice	mECM as a Scaffold	Enhanced chondrogenesis[44]
Porcine SM-MSCs	Porcine chondrocyte (passage 0, 2, and 6)	/	Greatly enhanced chondrocyte expansion but also delayed dedifferentiation of expanded chondrocytes[45]
Human BM-MSC	Human chondrocyte	/	promoted proliferation of chondrocytes and Chondrogenesis *in vitro* and *in vivo*[46]
Human AD-MSCs	immunodeficient NOD SCID gamma mice	/	Improved proliferation and induce chondrogenesis[47]

mECM origin	Seeded cell types and animal models	mECM Processing Methods	Function
Human AD-MSCs	Human AD-MSCs and immunocompetent CD1 (ICR)mice	solubilized mECM as a Hydrogel	Induced chondrogenesis without supplemental factors; formed hyaline cartilage-like tissue after *in vivo* implantation[48]
Rabbit BM-MSCs	Rabbit BM-MSCs and knee joint osteochondral defect model in rabbits	/	Increased chondrogenic differentiation potentia; significantly improved the regeneration of osteochondral defects in rabbits[6]
Human SM-MSCs (fetal and adult)	Human fetal SM-MSCs	/	adult SM-MSCs ECM enhanced chondrogenic potential of SM-MSCs[49]
Rabbit BM-MSCs	Rabbit BM-MSCs and nude mice	/	induced chondrogenic differentiation without any exogenous growth factors[50]
Rabbit BM-MSCs	Rabbit	lyophilization of mECM for scaffold fabrication	Greatly improved the quality of cartilage tissue *in vivo*[51]

Supplementary Table 5. Applications of mECMs for nerve/vascular regeneration

mECM origin	Seeded cell types and animal models	mECM Processing Methods	Function
Human BM-MSCs	Sciatic nerve defects in rats	mECM as a Scaffold	BM-MSCs ECM modified scaffolds facilitate the formation of myelin sheaths[52]
Human UC-MSCs	Sciatic nerve defects in rats	mECM as a Scaffold	Provide sufficient anchoring to promote axon regeneration and angiogenesis to achieve sciatic nerve repair[53]
Rat BM-MSCs	Sciatic nerve defects in rats	mECM as a Scaffold	Conducive to nerve regeneration[54]
Wharton's Jelly-derived mesenchymal stem cells	HUVECs	mECM as a scaffold coating	Promotes angiogenesis via integrin $\alpha V\beta 3$/c-Myc/P300/VEGF[55]
UC-MSCs	Schwann cell	mECM as a Scaffold	Promotes peripheral nerve repair[9]

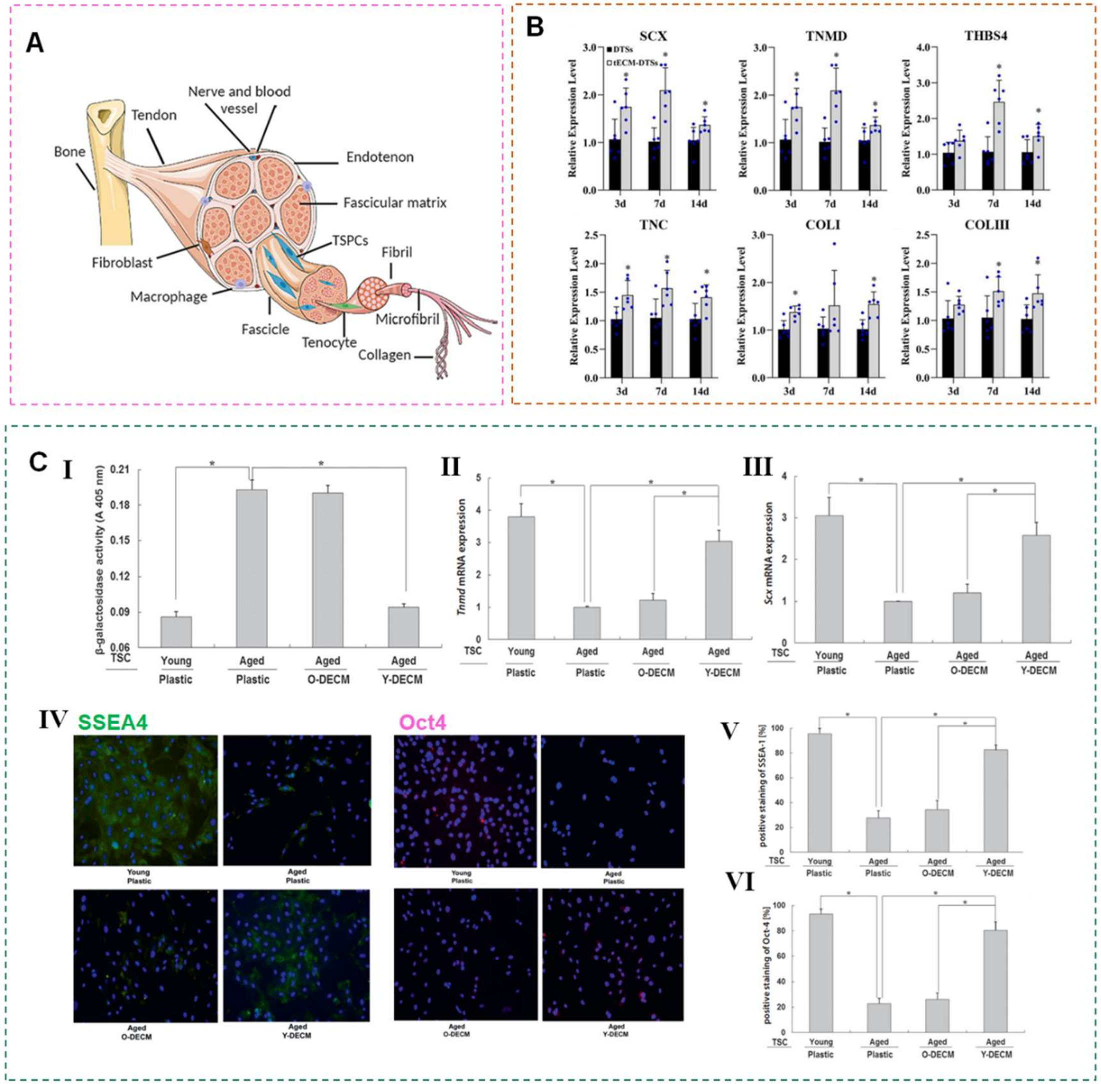

Supplementary Figure 1. Application of mECM in tendon.

A) Tendon hierarchical structure[56].

B) The mECM enhanced tenogenic differentiation[57].

C) I) The mECM reduces senescence-associated β-galactosidase (SA-β-gal) activity. II-III) The mECM enhanced tenogenic differentiation. IV-VI) The mECM promotes the maintenance of stemness in aged tendon stem cells[58].

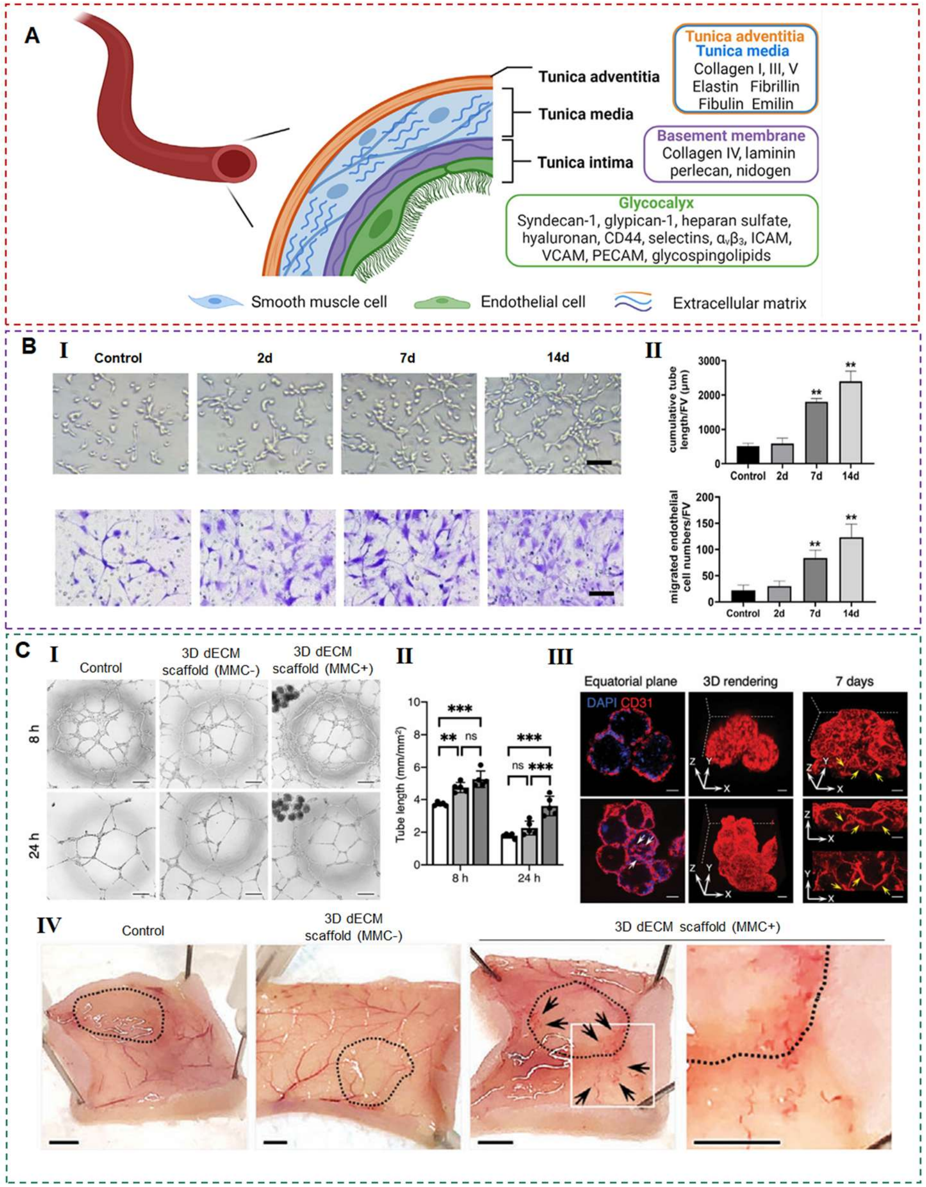

Supplementary Figure 2. Application of mECM in blood vessels.

A) Blood vessels hierarchical structure[59].

B) The mECM significantly promoted vascularization[55].

C) The 3D mECM spheroids remain bioactive *in vivo* and promote angiogenesis. I-II) The 3D mECM spheroids promote the formation of endothelial. III) The 3D mECM spheroids promotes the formation of the nascent tubular structures formed by HUVECs within the constructs. IV) The 3D mECM spheroids promote angiogenesis[60].

References

1. Hoshiba, T. *et al.* Effects of extracellular matrices derived from different cell sources on chondrocyte functions. *Biotechnol Prog* **27**, 788-795 (2011).
2. Mao, Y. *et al.* Extracellular matrix derived from chondrocytes promotes rapid expansion of human primary chondrocytes in vitro with reduced dedifferentiation. *Acta Biomater* **85**, 75-83 (2019).
3. Liu, C., Pei, M., Li, Q. & Zhang, Y. Decellularized extracellular matrix mediates tissue construction and regeneration. *Front Med* **16**, 56-82 (2022).
4. Sun, Y., Yan, L., Chen, S. & Pei, M. Functionality of decellularized matrix in cartilage regeneration: A comparison of tissue versus cell sources. *Acta Biomater* **74**, 56-73 (2018).
5. Shakouri-Motlagh, A., O'Connor, A. J., Brennecke, S. P., Kalionis, B. & Heath, D. E. Native and solubilized decellularized extracellular matrix: A critical assessment of their potential for improving the expansion of mesenchymal stem cells. *Acta Biomater* **55**, 1-12 (2017).
6. Wang, Z. *et al.* Extracellular matrix derived from allogenic decellularized bone marrow mesenchymal stem cell sheets for the reconstruction of osteochondral defects in rabbits. *Acta Biomater* **118**, 54-68 (2020).
7. Dikina, A. D., Almeida, H. V., Cao, M., Kelly, D. J. & Alsberg, E. Scaffolds Derived from ECM Produced by Chondrogenically Induced Human MSC Condensates Support Human MSC Chondrogenesis. *ACS Biomater Sci Eng* **3**, 1426-1436 (2017).
8. Lu, H., Hoshiba, T., Kawazoe, N. & Chen, G. Comparison of decellularization techniques for preparation of extracellular matrix scaffolds derived from three-dimensional cell culture. *J Biomed Mater Res A* **100**, 2507-2516 (2012).
9. Xiao, B. *et al.* Extracellular matrix from human umbilical cord-derived mesenchymal stem cells as a scaffold for peripheral nerve regeneration. *Neural Regen Res* **11**, 1172-1179 (2016).
10. Chen, X. D., Dusevich, V., Feng, J. Q., Manolagas, S. C. & Jilka, R. L. Extracellular matrix made by bone marrow cells facilitates expansion of marrow-derived mesenchymal progenitor cells and prevents their differentiation into osteoblasts. *J Bone Miner Res* **22**, 1943-1956 (2007).
11. Lai, Y. *et al.* Reconstitution of marrow-derived extracellular matrix ex vivo: a robust culture system for expanding large-scale highly functional human mesenchymal stem cells. *Stem Cells Dev* **19**, 1095-1107 (2010).
12. Pei, M., He, F. & Kish, V. L. Expansion on extracellular matrix deposited by human bone marrow stromal cells facilitates stem cell proliferation and tissue-specific lineage potential. *Tissue Eng Part A* **17**, 3067-3076 (2011).
13. He, F. *et al.* Extracellular matrix modulates the biological effects of melatonin in mesenchymal stem cells. *J Endocrinol* **223**, 167-180 (2014).
14. Zhou, L. *et al.* SIRT1-dependent anti-senescence effects of cell-deposited matrix on human umbilical cord mesenchymal stem cells. *J Tissue Eng Regen Med* **12**, e1008-e1021 (2018).
15. Liu, X. *et al.* Culturing on decellularized extracellular matrix enhances antioxidant properties of human umbilical cord-derived mesenchymal stem cells. *Mater Sci Eng C Mater Biol Appl* **61**, 437-448 (2016).

16. Li, M. *et al.* Inhibition of osteoclastogenesis by stem cell-derived extracellular matrix through modulation of intracellular reactive oxygen species. *Acta Biomater* **71**, 118-131 (2018).
17. Feng, Y. *et al.* Stem-cell-derived ECM sheet-implant complexes for enhancing osseointegration. *Biomater Sci* **8**, 6647-6656 (2020).
18. Larochette, N. *et al.* Osteogenic-differentiated mesenchymal stem cell-secreted extracellular matrix as a bone morphogenetic protein-2 delivery system for ectopic bone formation. *Acta Biomater* **116**, 186-200 (2020).
19. Sadr, N. *et al.* Enhancing the biological performance of synthetic polymeric materials by decoration with engineered, decellularized extracellular matrix. *Biomaterials* **33**, 5085-5093 (2012).
20. Hoch, A. I. *et al.* Cell-secreted matrices perpetuate the bone-forming phenotype of differentiated mesenchymal stem cells. *Biomaterials* **74**, 178-187 (2016).
21. Onishi, T. *et al.* Osteogenic extracellular matrix sheet for bone tissue regeneration. *Eur Cell Mater* **36**, 68-80 (2018).
22. Harvestine, J. N. *et al.* Extracellular Matrix-Coated Composite Scaffolds Promote Mesenchymal Stem Cell Persistence and Osteogenesis. *Biomacromolecules* **17**, 3524-3531 (2016).
23. Liao, J., Guo, X., Nelson, D., Kasper, F. K. & Mikos, A. G. Modulation of osteogenic properties of biodegradable polymer/extracellular matrix scaffolds generated with a flow perfusion bioreactor. *Acta Biomater* **6**, 2386-2393 (2010).
24. Thibault, R. A., Scott Baggett, L., Mikos, A. G. & Kasper, F. K. Osteogenic differentiation of mesenchymal stem cells on pregenerated extracellular matrix scaffolds in the absence of osteogenic cell culture supplements. *Tissue Eng Part A* **16**, 431-440 (2010).
25. Deng, M. *et al.* Modification of PLGA Scaffold by MSC-Derived Extracellular Matrix Combats Macrophage Inflammation to Initiate Bone Regeneration via TGF-beta-Induced Protein. *Adv Healthc Mater* **10**, e2100872 (2021).
26. Antebi, B. *et al.* Stromal-cell-derived extracellular matrix promotes the proliferation and retains the osteogenic differentiation capacity of mesenchymal stem cells on three-dimensional scaffolds. *Tissue Eng Part C Methods* **21**, 171-181 (2015).
27. Cai, R., Nakamoto, T., Kawazoe, N. & Chen, G. Influence of stepwise chondrogenesis-mimicking 3D extracellular matrix on chondrogenic differentiation of mesenchymal stem cells. *Biomaterials* **52**, 199-207 (2015).
28. Almeida, H. V. *et al.* Porous Scaffolds Derived from Devitalized Tissue Engineered Cartilaginous Matrix Support Chondrogenesis of Adult Stem Cells. *ACS Biomater Sci Eng* **3**, 1075-1082 (2017).
29. Ng, C. P. *et al.* Enhanced ex vivo expansion of adult mesenchymal stem cells by fetal mesenchymal stem cell ECM. *Biomaterials* **35**, 4046-4057 (2014).
30. Lu, H. *et al.* Cultured cell-derived extracellular matrix scaffolds for tissue engineering. *Biomaterials* **32**, 9658-9666 (2011).
31. Pizzute, T., Zhang, Y., He, F. & Pei, M. Ascorbate-dependent impact on cell-derived matrix in modulation of stiffness and rejuvenation of infrapatellar fat derived stem cells toward chondrogenesis. *Biomed Mater* **11**, 045009 (2016).
32. Li, J., He, F. & Pei, M. Creation of an in vitro microenvironment to enhance human fetal synovium-derived stem cell chondrogenesis. *Cell Tissue Res* **345**, 357-365 (2011).
33. Pei, M., Zhang, Y., Li, J. & Chen, D. Antioxidation of decellularized stem cell matrix promotes human synovium-derived stem cell-based chondrogenesis. *Stem Cells Dev* **22**,

889-900 (2013).
34. Li, J. *et al.* Rejuvenation of chondrogenic potential in a young stem cell microenvironment. *Biomaterials* **35**, 642-653 (2014).
35. Zhang, Y., Li, J., Davis, M. E. & Pei, M. Delineation of in vitro chondrogenesis of human synovial stem cells following preconditioning using decellularized matrix. *Acta Biomater* **20**, 39-50 (2015).
36. Zhang, Y., Pizzute, T., Li, J., He, F. & Pei, M. sb203580 preconditioning recharges matrix-expanded human adult stem cells for chondrogenesis in an inflammatory environment - A feasible approach for autologous stem cell based osteoarthritic cartilage repair. *Biomaterials* **64**, 88-97 (2015).
37. He, F., Chen, X. & Pei, M. Reconstruction of an in vitro tissue-specific microenvironment to rejuvenate synovium-derived stem cells for cartilage tissue engineering. *Tissue Eng Part A* **15**, 3809-3821 (2009).
38. Li, J. & Pei, M. Optimization of an in vitro three-dimensional microenvironment to reprogram synovium-derived stem cells for cartilage tissue engineering. *Tissue Eng Part A* **17**, 703-712 (2011).
39. Pei, M. *et al.* Repair of large animal partial-thickness cartilage defects through intraarticular injection of matrix-rejuvenated synovium-derived stem cells. *Tissue Eng Part A* **19**, 1144-1154 (2013).
40. He, F. & Pei, M. Extracellular matrix enhances differentiation of adipose stem cells from infrapatellar fat pad toward chondrogenesis. *J Tissue Eng Regen Med* **7**, 73-84 (2013).
41. Tang, C. *et al.* An autologous bone marrow mesenchymal stem cell-derived extracellular matrix scaffold applied with bone marrow stimulation for cartilage repair. *Tissue Eng Part A* **20**, 2455-2462 (2014).
42. Wang, Y. *et al.* Stem cells immortalized by hTERT perform differently from those immortalized by SV40LT in proliferation, differentiation, and reconstruction of matrix microenvironment. *Acta Biomater* **136**, 184-198 (2021).
43. Tang, C. *et al.* Evaluation of an Autologous Bone Mesenchymal Stem Cell-Derived Extracellular Matrix Scaffold in a Rabbit and Minipig Model of Cartilage Repair. *Med Sci Monit* **25**, 7342-7350 (2019).
44. Tang, C. *et al.* Feasibility of autologous bone marrow mesenchymal stem cell-derived extracellular matrix scaffold for cartilage tissue engineering. *Artif Organs* **37**, E179-190 (2013).
45. Pei, M. & He, F. Extracellular matrix deposited by synovium-derived stem cells delays replicative senescent chondrocyte dedifferentiation and enhances redifferentiation. *J Cell Physiol* **227**, 2163-2174 (2012).
46. Yang, Y. *et al.* Mesenchymal stem cell-derived extracellular matrix enhances chondrogenic phenotype of and cartilage formation by encapsulated chondrocytes in vitro and in vivo. *Acta Biomater* **69**, 71-82 (2018).
47. Yan, J. *et al.* Synovium stem cell-derived matrix enhances anti-inflammatory properties of rabbit articular chondrocytes via the SIRT1 pathway. *Mater Sci Eng C Mater Biol Appl* **106**, 110286 (2020).
48. Antich, C. *et al.* Development of a Biomimetic Hydrogel Based on Predifferentiated Mesenchymal Stem-Cell-Derived ECM for Cartilage Tissue Engineering. *Adv Healthc*

Mater **10**, e2001847 (2021).
49. Li, J., He, F. & Pei, M. Chondrogenic priming of human fetal synovium-derived stem cells in an adult stem cell matrix microenvironment. *Genes Dis* **2**, 337-346 (2015).
50. Tang, C., Jin, C., Xu, Y., Wei, B. & Wang, L. Chondrogenic Differentiation Could Be Induced by Autologous Bone Marrow Mesenchymal Stem Cell-Derived Extracellular Matrix Scaffolds Without Exogenous Growth Factor. *Tissue Eng Part A* **22**, 222-232 (2016).
51. Liu, J. *et al.* Cell-free scaffolds functionalized with bionic cartilage acellular matrix microspheres to enhance the microfracture treatment of articular cartilage defects. *J Mater Chem B* **9**, 1686-1697 (2021).
52. Wang, S. *et al.* Mechanisms underlying the cell-matrixed nerve grafts repairing peripheral nerve defects. *Bioact Mater* **31**, 563-577 (2024).
53. Guan, Y. *et al.* Dual-bionic regenerative microenvironment for peripheral nerve repair. *Bioact Mater* **26**, 370-386 (2023).
54. Wang, S. *et al.* BMSC-derived extracellular matrix better optimizes the microenvironment to support nerve regeneration. *Biomaterials* **280**, 121251 (2022).
55. Ma, B., Wang, T., Li, J. & Wang, Q. Extracellular matrix derived from Wharton's Jelly-derived mesenchymal stem cells promotes angiogenesis via integrin $\alpha V\beta 3$/c-Myc/P300/VEGF. *Stem Cell Res Ther* **13**, 327 (2022).
56. Zhu, G. *et al.* Bone physiological microenvironment and healing mechanism: Basis for future bone-tissue engineering scaffolds. *Bioact Mater* **6**, 4110-4140 (2021).
57. Ning, L. J. *et al.* Constructing a highly bioactive tendon-regenerative scaffold by surface modification of tissue-specific stem cell-derived extracellular matrix. *Regen Biomater* **9**, rbac020 (2022).
58. Jiang, D., Xu, B. & Gao, P. Effects of young extracellular matrix on the biological characteristics of aged tendon stem cells. *Adv Clin Exp Med* **27**, 1625-1630 (2018).
59. Jiang, S., Wise, S. G., Kovacic, J. C., Rnjak-Kovacina, J. & Lord, M. S. Biomaterials containing extracellular matrix molecules as biomimetic next-generation vascular grafts. *Trends Biotechnol* **42**, 369-381 (2024).
60. Chiang, C. E. *et al.* Bioactive Decellularized Extracellular Matrix Derived from 3D Stem Cell Spheroids under Macromolecular Crowding Serves as a Scaffold for Tissue Engineering. *Adv Healthc Mater* **10**, e2100024 (2021).
